# Nutrition Indicators, Physical Function, and Health-Related Quality of Life in Breast Cancer Patients

**DOI:** 10.31557/APJCP.2020.21.7.1939

**Published:** 2020-07

**Authors:** Krystal Ng Lu Shin, Chan Yoke Mun, Zalilah Mohd Shariff

**Affiliations:** 1 *Department of Nutrition and Dietetics, Faculty of Medicine and Health Sciences, Universiti Putra Malaysia, Malaysia. *; 2 *Department of Health Education, Literacy, Promotion and Policy, National Cancer Society of Malaysia, Malaysia. *; 3 *Research Centre of Excellence Nutrition and Non-communicable Diseases, Faculty of Medicine and Health Sciences, Universiti Putra Malaysia, Malaysia. *

**Keywords:** Breast cancer, Handgrip strength, health-related quality of life, nutritional status

## Abstract

**Objective:**

This study aimed to investigate how nutrition indicators and physical function may influence Health-related Quality of Life (HRQoL) of breast cancer patients undergoing treatment.

**Methods:**

This was a cross sectional study among a total of 163 breast cancer patients. Series of measurements including anthropometry, biochemical, and dietary were employed to assess patients’ nutritional status while physical function was assessed by handgrip strength. HRQoL of patients was determined using European Organization for Research and Treatment of Cancer quality of life questionnaire Core 30 (EORTC-QLQ-C30) version 3.0. Multiple linear regression was used to identify factors associated with HRQoL.

**Results:**

Breast cancer patients perceived moderately their overall quality of life (QoL), with the mean global health status (GHS) score of 69.12. Emotional functioning was the poorest functional scale while fatigue was the most distressing symptom presented by the patients. Approximately 20% of patients had low corrected arm muscle area while more than half had low hemoglobin level. More than 90% of patients did not meet the overall dietary recommendation and had poor handgrip strength. Mid-upper arm circumference (MUAC) was associated with GHS (β: 0.906; 95% CI: 0.22, 1.56) and cognitive functioning (β: -1.543; 95% CI: -3.07, -0.01). Handgrip strength was positively associated with most of HRQoL outcomes.

**Conclusions:**

Breast cancer patients reported overall good nutritional status and moderate QoL during treatment. Being well-nourished improved HRQoL and handgrip strength could be a potential proxy for functional outcomes as well as overall QoL.

## Introduction

A new paradigm for cancer care has been intensively deliberated, evolving from an aggressive cancer cure treatment to a life-prolonging treatment with the main consideration of Health-related quality of life (HRQoL) (Kelley and Meier, 2010; Patel et al., 2014). While HRQoL is gaining recognition as a prognostic indicator of cancer survival (Quinten et al., 2009) and has been equally acknowledged as an important clinical endpoint in cancer research (Fiteni et al., 2014), HRQoL outcomes should be emphasized in all cancer patients regardless of disease stages. It is essential for healthcare professionals to understand one’s perception of health or life status throughout cancer treatment as this could imply an effective clinical decision making, pertaining to better treatment adherence (Kane et al., 2014; Kelley and Meier, 2010). With improved management of functional disabilities or HRQoL impairment, a lower burden of hospitalization for cancer patients is anticipated.

Breast cancer population had received considerable attention in light of its high morbidity and mortality rates (Ferlay et al., 2014). Earlier detection awareness and advances in cancer treatment have greatly improved breast cancer survival rates, particularly in high-income countries (Torre et al., 2015). Nonetheless, treatment-related side effects on nutritional status such as nausea and vomiting, appetite loss and diarrhea, which negatively affect patients’ functional status and HRQoL (Dang et al., 2016) are commonly reported. While mounting evidence shows malnutrition is associated with decreasing HRQoL in cancer patients (Lis et al., 2012; Yu et al., 2013), the relationship between nutritional status and HRQoL is under-studied for breast cancer patients, who are at lower risk of under-nutrition as compared to other cancers (Lis et al., 2012). In spite of the negative impact of altered taste perception on calorie intake (Boltong et al., 2014; de Vries et al., 2017), weight gain was increasingly reported for breast cancer patients even during treatment (van den Berg et al., 2017), which may decrease their physical function and overall quality of life (QoL) (Fang et al., 2013), suggesting healthy eating habit for general health maintenance among the breast cancer patients is critical. Acknowledging nutrition disorders are modifiable determinants of health outcomes especially for impaired HRQoL, an effort to explore on the nutrition-related factors and cancer HRQoL is indispensable. On the other hand, primary treatment of axillary lymph node dissection can lead to muscle dysfunction and reduced limitation of doing routine activities (Hidding et al., 2014), elucidating the long term impairment of physical function and HRQoL. Investigating the association between muscle weakness and various aspects of functional health would enable a better understanding of the implication of physical limitation in overall QoL. 

Studies on HRQoL have been focusing among survivors, while the exploration of HRQoL determinants especially for breast cancer patients who receiving treatment is relatively scarce. This finding allows us to formulate more effective strategies to improve patients’ HRQoL at this critical period, which could assist the healthcare providers to make clinical decision wisely. This study utilizes the objective measure of handgrip strength to evaluate physical function, in order to minimize the self-report bias in HRQoL questionnaire. The parameters of anthropometric, biochemical, and diet quality were assessed to provide valuable information regarding the association of nutritional factors with HRQoL. Specifically, this study aimed to determine the handgrip strength and nutrition indicators of breast cancer patients in relation to HRQoL during treatment. 

## Materials and Methods

This was a cross sectional study. This study was conducted at National Cancer Institute, Putrajaya, Malaysia, a national referral center offering various treatments for optimum cancer care in Malaysia (Institut Kanser Negara, 2018), between January and May 2017. The samples were recruited from inpatient oncology wards and daycare center based on purposive sampling. Eligible patients were diagnosed with breast cancer, received at least one month of cancer treatment and were in the midway of receiving treatment. Patients were excluded if they were terminally ill, diagnosed with any psychiatric or neurological disorders. Ethics approval was provided by Medical Research and Ethics Committee (MREC), Ministry of Health Malaysia and Ethics Committee for Research Involving Human Subject Universiti Putra Malaysia. Patients’ written informed consents were obtained prior to study enrolment.

*Sociodemographic and medical characteristics*

Sociodemographic and medical characteristics of patients were retrieved from the hospital’s online information system, otherwise, via interview. Patients’ social support and physical activity level were assessed by the modified Medical Outcomes Study Social Support Survey (mMOS-SS) (Moser et al., 2012) and International Physical Activity Questionnaire-short form (IPAQ-SF) (Craig et al., 2003), respectively.

Biochemical data, including serum albumin, hemoglobin, and neutrophils count were obtained from the medical report as secondary data. Weight and height of patients were measured using Dectecto bariatric scale (6857DHR, Webb City, Missouri), with readings taken nearest to 0.1kg and 0.1cm, respectively. Weight status of patient was determined using the universal proxy, body mass index (BMI) according to international cut-off points (World Health Organization. Regional Office for the Western Pacific., 2000). Patients’ risk of malnutrition was determined by corrected arm muscle area (CAMA), which was computed from mid-upper arm circumference (MUAC) and triceps skinfold (TSF) thickness (Friedman et al., 1985). MUAC and TSF thickness were measured using Seca Ergonomic circumference measuring tape (201, Detuchland, Germany). With regards to evaluation of physical function, assessment on handgrip strength was ascertained at dominant hands of patients using Lafayette hand dynamometer (78010) and calssified based on age-specific criteria (Chen et al., 2014; Guerra et al., 2014). 

*Health-related quality of life*

Health-related quality of life (HRQoL) of patients was ascertained using European Organization for Research and Treatment of Cancer quality of life questionnaire Core 30 (EORTC-QLQ-C30) version 3.0. Being a widely used cancer-specific HRQoL questionnaire (Aaronson et al., 1993), EORTC-QLQ-C30 comprises of 30 items, which represents five functional scales (physical, role, emotional, cognitive, and social functioning), three symptom scales (fatigue, nausea, vomiting, and pain), a global health status scale and six single item symptoms (dyspnea, insomnia, appetite loss, constipation, diarrhea, and financial difficulties). Scoring on items of functional and symptoms scales were using four-point Likert scale, ranging from 1 (not at all) to 4 (very much), whereas a seven-point Likert scale was used to rate the items of global health status scale, ranging from 1 (very poor) to 7 (excellent). The mean of component items for each scale was linearly transformed into a range of 0 – 100 point, with higher scores indicate better functioning and QoL, but more severe symptoms (Fayers et al., 2001). 

*Healthy eating index (HEI) – 2015*

A 165 items Malaysian Semi quantitative food frequencies questionnaires (FFQ) was used to assess the habitual dietary intake of patients over the past one month (Institute for Public Health, 2014). Each food item was assigned a standard serving size according to Malaysian Food Album (Malaysian Food Album, 2011). The food consumption data was standardized to frequency consumption of daily basis. The Malaysian food composition database was primarily used for analyzing the nutrient intake via Nutritionist Pro version 4.0. 0 (Axxya system, 2017) with Singaporean and USDA database were the complementary data bases. To assess patients’ conformance to dietary guidelines, intakes of patients were transformed into Healthy Eating Index (HEI) scoring (Guenther et al., 2013). The intake of each food component was scored proportionately using the latest version of HEI-2015. The total HEI-2015 score was obtained by summing all individual component score, yielding a possible score range of 0 – 100, with higher scores indicate a better adherence to Americans’ dietary guidelines 2015-2020 (Guenther et al., 2013).

*Statistical analysis*

Data analysis was performed with Statistical Software Package (IBM SPSS statistics version 22.0). Mean and standard deviation were presented for normally distributed data. Multiple linear regression was performed to determine the contributions of handgrip strength and nutrition indicators (MUAC, TSF thickness, diet quality, serum albumin, hemoglobin level, and neutrophils count) to primary measure of global health status and other subscales according to potential confounding factors. Variables that were normally distributed for Global Health Status (GHS) and HRQoL subscales (with exception for dyspnea, diarrhea, nausea and vomiting) hence meeting the statistical assumption of using linear regression were selected in the analysis of Multiple Linear Regression. Two multivariate models were performed for each relationship of nutrition indicators and handgrip strength with a series of HRQoL subscales. The first model examined the associations of handgrip strength and each nutrition indicators with HRQoL subscales, adjusting for social support, moderate-to-vigorous physical activity, sociodemographic and medical characteristics. Model 2 examined if there were any changes of associations (resulted in Model 1) after mutually adjusted for nutrition indicators and handgrip strength in the model. All the significant values were set at p<0.05. 

## Results

*Study population*

Of 227 eligible patients approached, 179 patients were consented to participate in the study , giving a total response rate of 79%. In the analysis, 16 patients were excluded due to missing values (n=6) and extreme outliers (n=10). A total of 163 patients was included in the final analysis, constituting 69% of the approached sample.

*Subjects’ characteristics*

Details for sample are shown in Table 1. Mean age of patients was approximately 50 years (range, 29 - 71 years), with a mean duration of diagnosis at 8.66 months ± 5.96. More than half of the patients were diagnosed with advanced cancer (stage III: n=62; 38%; stage IV: n=41; 25.2%) and were currently receiving chemotherapy. The median MET score was 462 (IQR: 933), with majority of them were physically inactive. With regards to social support level, about half of the patients perceived their social support as average. 

Means weight and BMI were 61.78 kg ± 12.87 and 25.6 kg/m^2^ ± 5.13, respectively. Slightly more than half of the patients were overweight, with BMI≥25kg/m^2^. According to CAMA, an approximately 80% of patients were well-nourished. Means for serum albumin, neutrophils count, and hemoglobin level were 39.94 g/L ± 3.53, 3.81x10^9^/L ± 1.85 and 11.66 g/dL ± 1.20, respectively. Majority of patients had normal serum albumin (93.3%) and neutrophils count (81.6%), but with low hemoglobin level (57.1%). The mean of total HEI-2015 score was 63.86 ± 8.75. More than 90% of patients had poor diet quality or need dietary modification, with the scores below 80. Patients had mean handgrip strength of 9.60 kg ± 4.89, with as high as 98.2% of them had poor muscle strength.

*Health-related quality of life*

**Figure 1:**
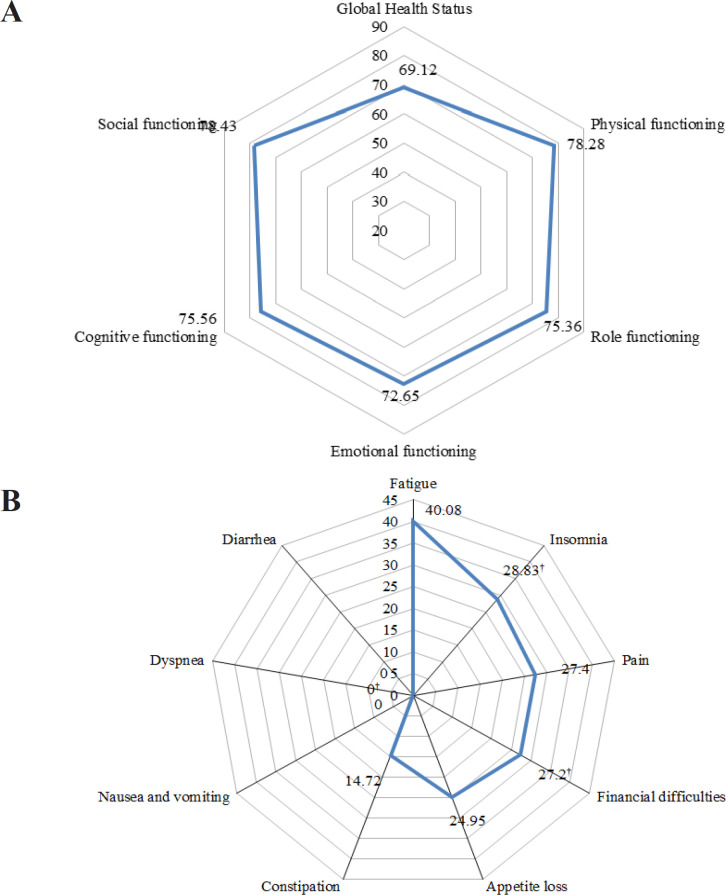
HRQoL Score According to Overall Global Health Status (A), sub-domains of functioning (A) and symptoms (B); †Median

As a measure for quality of life, the mean score of Global Health Status was 69.12. In term of functional scales, patients scored the highest for social and physical functioning but lowest for emotional functioning (Figure 1A). Among the nine medical symptoms, fatigue (40.08%) was most commonly experienced by patients, followed by insomnia (28.83%), pain (27.4%), financial difficulties (27.2%), and appetite loss (24.95%). Only a few patients reported nausea and vomiting, dyspnea, and diarrhea (Figure 1B). 

*Factors associated with Global Health Status*

The results of multiple linear regression models with cancer stage and active chemotherapy as categorical variables were illustrated in Table 2. Breast cancer patients with more advanced stage reported better Global Health Status than those in earlier stage. Patients who were undergoing active chemotherapy were more likely to have worse role function as compared to radiotherapy or other follow-up post-treatment. Tables 3, 4, and 5 demonstrated the multivariate associations of various nutrition indicators and handgrip strength with global health status, functional scales, and medical symptoms. For anthropometric indices, higher MUAC (β: 1.017; 95% CI: 0.35, 1.68) was related with better GHS after adjusting for social support, physical activity, sociodemographic and medical variables (Model 1). This association remained unchanged after mutually adjusted for nutrition indicators and handgrip strength (Model 2) (Table 2). On the other hand, MUAC was inversely associated with cognitive functioning after adjusting for all the covariates. Higher serum albumin was associated with improved physical functioning and pain (Model 2), but with worse insomnia (Table 3). A positive association of pain was shown with serum hemoglobin, only after controlling for handgrip strength and other nutrition indicators in addition to social support, physical activity, sociodemographic and medical variables (Model 2). Higher serum hemoglobin was associated with improved social functioning, fatigue, and constipation while neutrophils count was the only indicator which significantly associated with constipation (Table 3). Handgrip strength significantly associated with all HRQoL subscales with the exception on constipation and financial difficulties (Table 4).

**Table 1:** Participants’ Characteristics According to Sociodemographic, Medical, Social Support and Physical Activity Level (N=163)

	n (%)	Mean (SD)	Range
Age (years)		50.34 (10.22)	29.00 – 71.00
Marital status			
Single	16 (9.8)		
Married	112 (68.7)		
Widowed/Separated/Divorced	35 (21.5)		
Educational status			
No formal education or primary level	18 (11.0)		
Secondary level	80 (49.1)		
Tertiary level	65 (39.9)		
Being employed	65 (39.9)		
Monthly income (RM)		1995.66 (2163.68)	0 – 10, 000
Duration of diagnosis (months)		8.66 (5.96)	1.40 – 32.15
Cancer stage			
0 or I	6 (3.7)		
II	54 (33.1)		
III	62 (38.0)		
IV	41 (25.2)		
Treatment modalities (undergoing)			
Chemotherapy	104 (63.8)		
Radiotherapy	34 (20.9)		
Hormonal therapy	35 (21.5)		
Targeted therapy	25 (15.3)		
Have any comorbid condition	72 (44.2)		
Physical activity level (Total MET-min/week)		462 (933)†	0 – 8,200
Low	106 (65.0)		
Moderate	46 (28.2)		
High	11 (6.7)		
Level of social support		82.52 (17.26)	21.88 – 100.00
Low (1st quartile: <72)	44 (27.0)		
Medium (72 – 96)	86 (52.8)		
High (3rd quartile: ≥97)	33 (20.2)		
Weight (kg)		61.78 (12.87)	35.60 – 105.20
Height (m)		155.23 (5.48)	140.00 – 167.00
BMI (kg/m^2^)		25.63 (5.13)	14.92 – 42.70
Underweight (<18.50)	13 (8.0)		
Normal (18.50 – 24.99)	66 (40.5)		
Pre-obese (25.00 – 29.99)	50 (30.7)		
Obese (≥30.00)	34 (20.9)		
MUAC (cm)		29.93 (4.97)	18.80 – 44.95
TSF thickness (mm)		26.46 (7.84)	10.70 – 58.70
CAMA (cm^2^)		31.63 (12.26)	6.05 – 72.78
≤21.6	33 (20.2)		
>21.6	130 (79.8)		
Serum albumin (g/L)		39.94 (3.53)	28.00 – 50.00
Normal (35 – 50)	152 (93.3)		
Low (<35)	11 (6.7)		
	n (%)	Mean (SD)	Range
Neutrophils count (109/L)		3.81 (1.85)	0.13 – 10.24
Low (<2.00)	20 (12.3)		
Normal (2.00 – 7.00)	133 (81.6)		
High (>7.00)	10 (6.1)		
Hemoglobin level (g/dL)		11.66 (1.20)	9.00 – 15.70
Normal (12.00 – 15.00)	69 (42.3)		
Low (<12.00)	93 (57.1)		
Total HEI-2015 score		63.86 (8.75)	41.84 – 88.88
Good (>80)	7 (4.3)		
Needs improvement (51 – 80)	146 (89.6)		
Poor (<51)	10 (6.1)		
Handgrip strength (kg)		9.60 (4.89)	0.00 – 31.00
Poor	163 (98.2)		
Normal	3 (1.8)		

Data were presented as n (%) or mean (SD); Ringgit Malaysia exchange rate: 1 MYR, 0.25 USD; Cancer stage: based on anatomic and histological evaluation; †Median (IQR); BMI, body mass index; CAMA, corrected arm muscle area; MUAC, mid-upper arm circumference; TSF thickness, triceps skinfold thickness; HEI, healthy eating index

**Table 2:** Multivariate Associations of Cancer Stage and Active Chemotherapy with 12 HRQoL Scales

Nutritional parameters	Cancer stage (III/IV vs 0/I/II)			Active chemotherapy (Yes vs No)		
	β	*P*-value	95%CI	β	*P*-value	95%CI
Global Health Status						
Model 1	7.999	0.014	(1.65;14.35)	-2.019	0.572	(-9.06; 5.02)
Model 2	6.221	0.044	(0.17, 12.28)	0.449	0.896	(-6.34; 7.24)
Functional scales						
Physical						
Model 1	-3.647	0.245	(-9.82; 2.52)	-1.887	0.587	(-8.73; 4.96)
Model 2	-5.022	0.064	(-10.35; 0.30)	-1.583	0.601	(-7.55; 4.39)
Role						
Model 1	-5.832	0.268	(-16.20; 4.54)	-11.795	0.045	(-23.30; -0.29)
Model 2	-6.991	0.179	(-17.22; 3.24)	-11.681	0.046	(-23.14; -0.22)
Emotional						
Model 1	3.216	0.448	(-5.15; 11.58)	5.245	0.266	(-4.03; 14.52)
Model 2	1.164	0.785	(-7.24; 9.57)	7.505	0.117	(-1.91; 16.93)
Cognitive						
Model 1	4.394	0.34	(-4.68; 13.47)	-1.173	0.818	(-11.24; 8.90)
Model 2	2.857	0.537	(-6.27; 11.98)	-1.109	0.831	(-11.34; 9.12)
Social						
Model 1	-3.295	0.491	(-12.73; 6.14)	-8.391	0.115	(-18.85; 2.07)
Model 2	-5.168	0.274	(-14.48; 4.14)	-4.642	0.381	(-15.07; 5.79)
Symptoms scales/items						
Fatigue						
Model 1	-3.116	0.441	(-11.08; 4.85)	7.638	0.09	(-1.20; 16.47)
Model 2	-2.789	0.485	(-10.66; 5.08)	5.431	0.226	(-3.39; 14.25)
Pain						
Model 1	-1.327	0.793	(-11.32; 8.67)	-0.448	0.937	(-11.54; 10.64)
Model 2	0.17	0.972	(-9.42; 9.76)	0.329	0.952	(-10.42; 11.08)
Insomnia						
Model 1	-1.827	0.77	(-14.16; 10.51)	-7.894	0.256	(-21.58; 5.79)
Model 2	0.718	0.908	(-11.53; 12.97)	-6.834	0.327	(-20.57; 6.90)
Appetite loss						
Model 1	-5.626	0.31	(-16.53; 5.28)	9.688	0.116	(-2.41; 21.79)
Model 2	-5.604	0.31	(-16.48; 5.27)	9.127	0.141	(-3.06; 21.31)
Constipation						
Model 1	0.128	0.975	(-7.89; 8.15)	0.19	0.966	(-8.71; 9.09)
Model 2	1.452	0.719	(-6.50; 9.40)	-3.497	0.439	(-12.41; 5.41)
Financial difficulties						
Model 1	-0.085	0.988	(-11.54; 11.37)	-4.552	0.48	(-17.26; 8.16)
Model 2	0.706	0.907	(-11.19; 12.60)	-4.967	0.463	(-18.30; 8.36)

Model 1, Adjusted for age, being employed, monthly income, married, educational level (secondary, tertiary or primary, no formal education) + cancer stage (III, IV or 0, I, II), time since diagnosis, chemotherapy, comorbidity + social support + moderate/vigorous physical activity level; Model 2, Model 1 + nutrition indicators + handgrip strength Figures in bold indicate statistically significant values (p<0.05)

**Table 3:** Multivariate Associations of Anthropometric and Dietary Indicators with 12 HRQoL Scales

Nutritional parameters	MUAC			TSF thickness			HEI-2015		
	β	*P-*value	95%CI	β	*P*-value	95%CI	β	*P*-value	95%CI
Global Health Status									
Model 1	1.017	0.003	(0.35, 1.68)	0.241	0.254	(-0.18, 0.66)	-0.124	0.51	(-0.49, 0.25)
Model 2	1.277	0.014	(0.27, 2.29)	-0.356	0.247	(-0.96, 0.25)	-0.095	0.597	(-0.45, 0.26)
Functional scales									
Physical									
Model 1	0.156	0.642	(-0.51, 0.82)	0.127	0.535	(-0.28, 0.53)	0.264	0.145	(-0.09, 0.62)
Model 2	-0.332	0.465	(-1.23, 0.56)	0.347	0.203	(-0.19, 0.88)	0.202	0.206	(-0.11, 0.52)
Role									
Model 1	-0.372	0.5	(-1.46, 0.72)	-0.17	0.613	(-0.84, 0.49)	0.209	0.484	(-0.38, 0.80)
Model 2	-0.957	0.261	(-2.64, 0.72)	0.338	0.508	(-0.67, 1.34)	0.07	0.814	(-0.52, 0.66)
Emotional									
Model 1	-0.395	0.387	(-1.30, 0.51)	-0.493	0.076	(-1.04, 0.05)	0.016	0.947	(-0.47, 0.50)
Model 2	0.222	0.756	(-1.19, 1.63)	-0.629	0.144	(-1.48, 0.22)	0.058	0.816	(-0.44, 0.55)
Cognitive									
Model 1	-0.715	0.146	(-1.68, 0.25)	-0.177	0.557	(-0.77, 0.42)	-0.194	0.466	(-0.72, 0.33)
Model 2	-1.543	0.048	(-3.07, -0.01)	0.553	0.235	(-0.36, 1.47)	-0.283	0.299	(-0.82, 0.25)
Social									
Model 1	0.112	0.827	(-0.90, 1.23)	0	0.999	(-0.62, 0.62)	-0.348	0.21	(-0.89, 0.20)
Model 2	-0.231	0.77	(-1.79, 1.33)	0.083	0.862	(-0.85, 1.02)	-0.497	0.075	(-1.05, 0.05)
Symptoms scales/items									
Fatigue									
Model 1	0.469	0.278	(-0.38, 1.32)	0.42	0.11	(-0.10, 0.94)	-0.41	0.077	(-0.87, 0.05)
Model 2	0.017	0.979	(-1.29, 1.33)	0.402	0.313	(-0.38, 1.19)	-0.298	0.202	(-0.76, 0.16)
Pain									
Model 1	-0.389	0.472	(-1.46, 0.68)	-0.32	0.332	(-0.97, 0.33)	-0.45	0.122	(-1.02, 0.12)
Model 2	0.221	0.786	(-1.38, 1.82)	-0.476	0.329	(-1.44, 0.49)	-0.491	0.087	(-1.06, 0.07)
Insomnia									
Model 1	-0.814	0.224	(-2.13, 0.50)	-0.28	0.493	(-1.09, 0.53)	0.012	0.974	(-0.70, 0.73)
Model 2	-0.235	0.82	(-2.27, 1.80)	-0.152	0.807	(-1.37, 1.07)	-0.199	0.584	(-0.92, 0.52)
Appetite loss									
Model 1	-0.625	0.287	(-1.78, 0.53)	-0.34	0.343	(-1.05, 0.37)	-0.523	0.099	(-1.14, 0.10)
Model 2	-0.413	0.652	(-2.22, 1.39)	-0.206	0.707	(-1.29, 0.88)	-0.444	0.169	(-1.08, 0.19)
Constipation									
Model 1	0.252	0.565	(-0.61, 1.12)	0.098	0.713	(-0.43, 0.63)	0.153	0.517	(-0.31, 0.62)
Model 2	0.128	0.849	(-1.20, 1.46)	0.11	0.786	(-0.69, 0.91)	0.206	0.385	(-0.26, 0.67)
Financial difficulties									
Model 1	0.297	0.635	(-0.94, 1.53)	-0.022	0.954	(-0.78, 0.73)	0.207	0.541	(-0.46, 0.87)
Model 2	0.852	0.401	(-1.15, 2.85)	-0.372	0.541	(-1.57, 0.83)	0.212	0.552	(-0.49, 0.91)

Model 1, Adjusted for age, being employed, monthly income, married, educational level (secondary, tertiary, or primary or no formal education) + cancer stage (III, IV, or 0, I or II), time since diagnosis, chemotherapy, comorbidity + social support + moderate/vigorous physical activity level; Model 2, Model 1 + nutrition indicators + handgrip strength Figures in bold indicate statistically significant values (p<0.05)

**Table 4:** Multivariate Associations of Biochemical Indicators with 12 HRQoL Scales

Nutritional parameters	Serum albumin			Serum hemoglobin			Neutrophils count		
	β	*P-*value	95%CI	β	*P-*value	95%CI	β	*P-*value	95%CI
Global Health Status									
Model 1	-0.182	0.761	(-1.05, 0.77)	1.217	0.357	(-1.39, 3.82)	0.527	0.539	(-1.16, 2.22)
Model 2	-0.133	0.765	(-1.02, 0.75)	1.391	0.292	(-1.21, 4.00)	0.671	0.418	(-0.96, 2.31)
Functional scales									
Physical									
Model 1	1.626	<0.001	(0.80, 2.45)	0.037	0.977	(-2.50, 2.57)	-0.198	0.811	(-1.84, 1.44)
Model 2	1.694	<0.001	(0.91, 2.48)	-2.174	0.064	(-4.47, 0.13)	0.484	0.509	(-0.96, 1.93)
Role									
Model 1	1.143	0.112	(-0.27, 2.56)	0.515	0.807	(-3.64, 4.67)	-1.358	0.319	(-4.04, 1.33)
Model 2	0.927	0.213	(-0.54, 2.39)	-0.754	0.73	(-5.07, 3.56)	-1.012	0.462	(-3.73, 1.70)
Emotional									
Model 1	-0.303	0.613	(-1.48, 0.88)	0.287	0.869	(-3.16, 3.73)	1.451	0.198	(-0.77, 3.67)
Model 2	-0.328	0.599	(-1.56, 0.90)	0.109	0.953	(-3.52, 3.73)	1.793	0.122	(-0.49, 4.07)
Cognitive									
Model 1	0.761	0.237	(-0.51, 2.03)	-0.44	0.815	(-4.15, 3.27)	0.241	0.843	(-2.16, 2.65)
Model 2	0.713	0.292	(-0.62, 2.05)	-1.381	0.488	(-5.31, 2.55)	0.183	0.884	(-2.29, 2.06)
Social									
Model 1	0.321	0.633	(-1.01, 1.65)	4.269	0.028	(0.46, 8.08)	0.348	0.784	(-2.16, 2.85)
Model 2	-0.099	0.885	(-1.46, 1.26)	4.615	0.024	(0.61, 8.63)	-0.214	0.867	(-2.74, 2.31)
Symptoms scales/items									
Fatigue									
Model 1	-0.644	0.254	(-1.76, 0.47)	-3.554	0.03	(-6.76, -0.35)	0.351	0.743	(-1.76, 2.46)
Model 2	-0.174	0.764	(-1.32, 0.97)	-2.963	0.084	(-6.37, 0.40)	0.103	0.923	(-2.01, 2.22)
Pain									
Model 1	-1.377	0.05	(-2.76, 0.00)	1.452	0.482	(-2.62, 5.52)	-0.993	0.457	(-3.63, 1.64)
Model 2	-1.672	0.019	(-3.07, -0.27)	4.285	0.042	(0.17, 8.40)	-1.987	0.132	(-4.58, 0.60)
Insomnia									
Model 1	1.837	0.035	(0.14, 3.54)	4.969	0.05	(-0.01, 9.95)	-1.43	0.387	(-4.69, 1.83)
Model 2	1.37	0.13	(-0.41, 3.15)	4.79	0.073	(-0.44, 10.03)	-1.921	0.251	(-5.22, 1.37)
Appetite loss									
Model 1	-1.444	0.059	(-2.94, 0.06)	-2.472	0.27	(-6.88, 1.94)	0.605	0.677	(-2.26, 3.47)
Model 2	-1.285	0.109	(-2.86, 0.29)	-0.7	0.766	(-5.34, 3.94)	0.064	0.965	(-2.85, 2.98)
Constipation									
Model 1	-0.529	0.355	(-1.66, 0.60)	-4.609	0.005	(-7.82, -1.40)	-2.761	0.01	(-4.85, -0.68)
Model 2	-0.246	0.676	(-1.41, 0.92)	-4.053	0.021	(-7.48, -0.63)	-2.43	0.027	(-4.58, -0.28)
Financial difficulties									
Model 1	0.287	0.726	(-1.33, 1.90)	-0.543	0.82	(-5.26, 4.17)	-1.526	0.323	(-4.57, 1.52)
Model 2	0.352	0.691	(-1.39, 2.09)	-0.53	0.838	(-5.66, 4.60)	-1.164	0.477	(-4.39, 2.07)

Model 1, Adjusted for age, being employed, monthly income, married, educational level (secondary, tertiary, or primary or no formal education) + cancer stage (III, IV, or 0, I or II), time since diagnosis, chemotherapy, comorbidity + social support + moderate/vigorous physical activity level; Model 2, Model 1 + nutrition indicators + handgrip strength Figures in bold indicate statistically significant values (p<0.05)

**Table 5:** Multivariate Associations of Handgrip Strength with 12 HRQoL Scales

	Handgrip strength		
	β	*P-*value	95%CI
Global Health Status			
Model 1	1.382	<0.001	(0.77, 2.00)
Model 2	1.215	<0.001	(0.59, 1.84)
Functional scales			
Physical			
Model 1	1.751	<0.001	(1.18, 2.32)
Model 2	1.782	<0.001	(1.23, 2.34)
Role			
Model 1	1.788	0.001	(0.79, 2.79)
Model 2	1.88	<0.001	(0.84, 2.92)
Emotional			
Model 1	1.038	0.017	(0.19,1.89)
Model 2	1.031	0.021	(0.16, 1.91)
Cognitive			
Model 1	0.877	0.062	(-0.04, 1.80)
Model 2	1.11	0.022	(0.16, 2.06)
Social			
Model 1	1.441	0.003	(0.50, 2.38)
Model 2	1.447	0.004	(0.48, 2.41)
Symptoms scales/items			
Fatigue			
Model 1	-1.164	0.004	(-1.96, -0.37)
Model 2	-1.092	0.009	(-1.90, -0.28)
Pain			
Model 1	-1.703	0.001	(-2.69, -0.72)
Model 2	-1.761	0.001	(-2.75, -0.77)
Insomnia			
Model 1	-1.61	0.011	(-2.85, -0.37)
Model 2	-1.705	0.008	(-2.97, -0.44)
Appetite loss			
Model 1	-1.423	0.011	(-2.51, -0.34)
Model 2	-1.303	0.023	(-2.42, -0.19)
Constipation			
Model 1	-0.49	0.241	(-1.31, 0.33)
Model 2	-0.509	0.225	(-1.33, 0.32)
Financial difficulties			
Model 1	-0.13	0.828	(-1.31, 1.05)
Model 2	-0.289	0.645	(-1.53, 0.95)

Model 1, Adjusted for age, being employed, monthly income, married, educational level (secondary, tertiary, or primary or no formal education) + cancer stage (III, IV, or 0, I or II), time since diagnosis, chemotherapy, comorbidity + social support + moderate/vigorous physical activity level; Model 2, Model 1 + nutrition indicators Figures in bold indicate statistically significant values (p<0.05)

## Discussion

As nutritional status was a determinant for HRQoL (Lis et al., 2012), present study highlighted the importance of a comprehensive nutritional assessment among breast cancer patients in light of its associations with QoL, functional health, and medical symptoms. Most of the nutrition indicators especially BMI indicated that majority of breast cancer patients were over-nourished. This finding was in agreement with previous studies (Custódio et al., 2016; Fang et al., 2013), which is likely due to inactive lifestyle resulted in energy imbalance (Vance et al., 2011), partially attributed to the high prevalence of physically inactive among the patients. More studies are warranted as present study did not determine the changes of physical activity level before and during treatment. With respect to cancer severity, a study revealed a total of 51.4%, 48.8%, and 46.1% of breast cancer patients with stage I, II, and III, respectively practiced moderate-to-vigorous physical activity level (20.2 MET-hours) during treatment, however, the strength of association between cancer stage and physical activity was weak (Mandelblatt et al., 2011). It is reasonable that overweight in breast cancer stem from climate factor (Von Hippel and Benson, 2014), as this study was conducted during hot session which may discourage the patients to carry out physical activity, especially outdoor activities. On the other hand, hemoglobin level was slightly lower than the recommendation. In addition to nutritional issues, the abnormally low hemoglobin level could be manifested by disease- or treatment-related factors (Hidding et al., 2014; Naoum, 2016), elucidating a low specificity in respect of the nutritional prognosis. Therefore, a multimodal of nutritional assessment should be taken into consideration to ensure diagnostic accuracy.

With regards to HRQoL pattern, present study indicated an average score of overall QoL, which is comparable with previous studies (Høyer et al., 2011; Ng et al., 2015; Rohani et al., 2015). On the other hand, varied distressing symptoms and functional limitations were widely reported across the studies (Høyer et al., 2011; Ng et al., 2015; Rohani et al., 2015). Discrepancies of HRQoL between countries could be explained by the variation in social cultural perspective as well as the economy status. The scores for certain symptoms among the patients in this study were zero when compared to the EORTC-QLQ-C30 population reference values (Scott et al., 2008). Likewise, nausea and vomiting, dyspnea, and diarrhea were the least complaint symptoms in present study, which explained the asymmetric radar chart. It should be noted that HRQoL is highly dependent on personal values or priorities, whereby each domain could be perceived differently across individual as well as population. 

Unexpectedly, patients with higher grade of breast cancer perceived better overall QoL during treatment. The result of Iran study revealed that breast cancer patients with more advanced cancer were likely to experience worse QoL, in the aspects of physical and role functioning (Rohani et al., 2015). With the continuity of active treatment, a majority of patients especially those diagnosed with advanced cancer received palliative care for symptom relief purpose (Kelley and Meier, 2010). This may elucidate the benefit of initiating palliative care for cancer HRQoL while receiving aggressive cancer treatments. In comparison with other treatments including of radiotherapy, hormonal therapy, and targeted therapy, chemotherapy resulted in poorer role functioning among breast cancer patients. Chemotherapy drugs attack the cancer cells by entering the blood circulation, which could also damage the healthy cells, resulting in symptoms of altered appetite, nausea and vomiting (American Cancer Society, 2016), which could affect patients’ ability to perform daily activities and their HRQoL.

With the exceptions of constipation and financial difficulties, higher handgrip strength was consistently associated with better HRQoL. Highly related with impaired physical function and overall QoL (Christensen et al., 2014; Kilgour et al., 2013), impaired muscle function is evident among breast cancer patients (Hidding et al., 2014), which is attributable to lymph node dissection or cancer treatment (Hidding et al., 2014). The assessment of handgrip strength reflects well the strength of arm muscles, in relation to physical function. Despite the utilization of handgrip strength remains low in clinical setting, it can be considered as a good proxy for physical function evaluation, which is recognized as one of the major functional components for HRQoL evaluation. Handgrip strength shows incompatible result with body composition indices in regard to the evaluation of nutritional status, elucidating that it is a relatively weak indicator of muscle wasting or malnutrition (Shi and Chen, 2017) for breast cancer patients during treatment. Benavides-Rodríguez et al., (2017) had previously demonstrated an inverse association between handgrip strength and body mass index in breast cancer survivors (Benavides-Rodríguez et al., 2017). More studies are needed to delineate the significant role of handgrip strength measurement among this population, particularly on-treatment phase.

On the other hand, all nutrition indicators except TSF thickness and diet quality were found to be associated significantly with various aspects of HRQoL. In corroborating with previous studies (Rahman et al., 2014; Xia et al., 2018), current finding revealed that overall QoL was improved with MUAC, which is a good surrogate of BMI (Brito et al., 2016). As MUAC and BMI have strong multicollinearity, MUAC that explained better the HRQoL outcomes was selected in the final regression model. Earlier systematic review supported the relationship of HRQoL with nutritional status (Lis et al., 2012). Treatment-induced side effects predispose cancer patients to poor oral intake and under-nutrition, which negatively affect their HRQoL, particularly for physical and emotional function (Arends et al., 2017). However, the direction of relationship between HRQoL and nutritional status remains unanswered. Studies on breast cancer demonstrated negative impact of being obesity on HRQoL (Doll et al., 2015; Fang et al., 2013; Paxton et al., 2012), where poorer HRQoL was found with higher body fat level (Frenzel et al., 2013). As seen in the present study, reduced cognitive function was shown as MUAC increases, which could be explained by the physiology of obesity induced cognitive impairment (O’Brien et al., 2017). As there is little evidence about the relationship of body composition with HRQoL, more studies are deemed necessary to prove this notion. It is noteworthy that muscle mass and body fat percentage of breast cancer patients were assessed. Inconsistent with previous studies (George et al., 2014; Wayne et al., 2006), diet quality did not show any significant finding with HRQoL. This could be due to different instruments used to assess diet quality and HRQoL.

All biochemical indicators were shown to contribute to better HRQoL, except for insomnia and pain scales. Although higher serum albumin was significantly associated with worse insomnia, this relationship became attenuated when controlling for nutrition indicators and handgrip strength (Model 2). This suggests that nutrition indicators or handgrip strength could be potential confounders of the relationship between serum albumin and insomnia. Romero-Corral et al., (2010) proposed that being obesity is the major contributor of obstructive sleep apnea, as manifested by symptom of insomnia (Romero-Corral et al., 2010). Blood transfusion is commonly used to manage cancer-related anemia, which may explain the finding of positive relationship between serum hemoglobin and pain. A result of breast cancer study revealed that higher serum hemoglobin resulted in improved fatigue and functional capacity (Jacobsen et al., 2004), which was in line with present study. Hemoglobin is a type of protein that carry and supply oxygen for cellular activities, which related closely with fatigue. In view of the interrelation among symptoms, functional health, and overall QoL (Ferrans et al., 2005), present finding suggests the possible negative impact of low serum hemoglobin on social functioning by inducing fatigue. As shown in the finding, patients with low neutrophils count were likely to experience constipation. An abnormally low neutrophils count should be paid with great attention as it might indicate higher risk of getting constipation induced-infection, which could be life-threatening (Kawsar et al., 2012). There was a substantial proportion of patients diagnosed with high graded cancer and receiving chemotherapy, reflecting the studied population are weak or vulnerable. As disease and treatment-related factors pose substantially effect on patients’ HRQoL, the main essence of study in determining the association between nutrition indicators and physical function with HRQoL could be affected. Despite these two factors were controlled for multivariate analysis, the finding should be interpreted cautiously. 

The strengths of the present study include the exploration of nutrition indicators, which were in relation to multi-aspects of HRQoL, accounting for various potential confounders. However, as this study was a cross sectional design in nature, hence the causality of association between nutritional factors and HRQoL could not be confirmed. Lack of control group as a point of comparison may diminish the ability to identify the extent to which breast cancer patients perceived their HRQoL during treatment, whereby a case control study should be initiated in the future. On the other hand, as this study recruited patients from a single institution, the generalization of study population could not be precluded. There is possibility of eliminating patients with emotional distress or impaired HRQoL, given that the participation of this study was on voluntary basis. Moreover, the current finding may underestimate the symptoms induced by cancer treatment due to large gap between treatment and assessment days. 

The physical measure of handgrip strength was the most prominent indicator for most of the HRQoL outcomes. Handgrip strength is considered as the recognized measure used for HRQoL evaluation, particularly in the aspect of physical function. A good nutritional status was associated with improved overall QoL. Current finding depicts potential benefits of being well-nourished and having stronger muscle on HRQoL outcomes among breast cancer patients undergoing treatment, which highlights the need for appropriate nutritional intervention to improve HRQoL among breast cancer patients.
